# Differentiation of pancreatic carcinoma and mass-forming focal pancreatitis: qualitative and quantitative assessment by dynamic contrast-enhanced MRI combined with diffusion-weighted imaging

**DOI:** 10.18632/oncotarget.12120

**Published:** 2016-09-19

**Authors:** Ting-Ting Zhang, Li Wang, Huan-huan Liu, Cai-yuan Zhang, Xiao-ming Li, Jian-ping Lu, Deng-bin Wang

**Affiliations:** ^1^ Department of Radiology, Xinhua Hospital, Shanghai Jiao Tong University School of Medicine, Shanghai, China; ^2^ Department of Radiology, Changhai Hospital, Second Military Medical University, Shanghai, China

**Keywords:** pancreatic carcinoma, mass-forming focal pancreatitis, dynamic contrast-enhanced MRI, diffusion-weighted imaging

## Abstract

Differentiation between pancreatic carcinoma (PC) and mass-forming focal pancreatitis (FP) is invariably difficult. For the differential diagnosis, we qualitatively and quantitatively assessed the value of dynamic contrast-enhanced MRI (DCE-MRI) and diffusion-weighted imaging (DWI) in PC and FP in the present study. This study included 32 PC and 18 FP patients with histological confirmation who underwent DCE-MRI and DWI. The time-signal intensity curve (TIC) of PC and FP were classified into 5 types according to the time of reaching the peak, namely, type I, II, III, IV, and V, respectively, and two subtypes, namely, subtype-a (washout type) and subtype-b (plateau type) according to the part of the TIC profile after the peak. Moreover, the mean and relative apparent diffusion coefficient (ADC) value between PC and FP on DWI were compared. The type V TIC was only recognized in PC group (*P* < 0.01). Type IV b were more frequently observed in PC (*P* = 0.036), while type- IIa (*P* < 0.01), type- Ia (*P* = 0.037) in FP. We also found a significant difference in the mean and relative ADC value between PC and FP. The combined image set of DCE-MRI and DWI yielded an excellent sensitivity, specificity, and diagnostic accuracy (96.9%, 94.4%, and 96.0%). The TIC of DCE-MRI and ADC value of DWI for pancreatic mass were found to provide reliable information in differentiating PC from FP, and the combination of DCE-MRI and DWI can achieve a higher sensitivity, specificity, and diagnostic accuracy.

## INTRODUCTION

The accurate diagnosis of mass-forming focal pancreatitis (FP) is extremely critical because FP has a different prognosis and treatment strategy in comparison with pancreatic carcinoma (PC) [1–3]. Surgical resection provides the treatment of choice for patients with PC; however, this process may bring about the risk to the patients with FP resulting in certain mortality [4]. Moreover, the differential diagnosis is invariably problematic since PC and FP may have mimicking clinical presentations and imaging findings, and even biopsy remains inconclusive [5–7].

Currently, magnetic resonance imaging (MRI) of the pancreas has increasingly been used as the primary imaging study of choice [5]. With advances in imaging technologies including diffusion-weighted imaging (DWI), MRI can improve detection and characterization of pancreatic lesions, as well as staging of tumors and inflammation [8]. However, the rate of misdiagnosis in differentiation between PC and FP is as high as 25% [9]. Dynamic contrast-enhanced MRI (DCE-MRI) and diffusion-weighted imaging (DWI) have been widely implemented as prominent imaging techniques. Pancreatic time-signal intensity curve (TIC) from DCE-MRI was revealed to provide valuable data for differentiating PC from FP and also for detecting the PC associated with chronic pancreatitis [10]. DWI detects random water motion within vital tissues and produces a representative apparent diffusion coefficient (ADC) value. ADC values have been already utilized to discriminate benign from malignant lesions for the abdominal diseases and ADC values of malignant pancreatic lesions are remarkably lower than those of benign ones [11, 12].

Although previous studies have already identified some different findings between PC and FP [13–16], they only evaluated the utility of a single technique, and the sensitivity of any single technique was not very high, while the combined technical approach offered a higher specificity. Higher sensitivity is warranted in order to preclude unnecessary pancreatic surgeries. Therefore, we evaluated the diagnostic accuracy of DCE-MRI and DWI findings, respectively, and combined ability for differentiation of PC from FP based on qualitative and quantitative analysis of TIC and ADC values. Additionally, the relative values of the mass to non-mass adjacent pancreatic parenchyma (NAP) were further investigated.

## RESULTS

### Patients

The frequencies of Patient characteristics, clinical presentations, and MRI features were listed in Table 1. Histopathologic analysis of the 32 PC patients indicated that 25 tumors arose from the pancreatic head, 3 from the neck, and 4 from the body/tail. In FP patients, 14 lesions arose from the pancreatic head and 11 from the pancreatic body/tail. The clinical stage and pathological grade of pancreatic cancer were presented in Table 2. Clinical TNM classification was performed according to American Joint Committee on Cancer (AJCC) Staging System, based on the combined data of the tumor acquired during surgery, the histological inspection or imaging studies. The underlying causes of 18 FP included alcoholic (*n* = 10), idiopathic (*n* = 3), autoimmune (*n* = 3), gallstone (*n* = 1), and pancreatic divisum (*n* = 1).

**Table 1:** Patient MRI features and laboratory data of 32 PC and 18 FP lesions

	PC (N, %)	FP (N, %)	*P* value	K value
Location			0.306	1
Head	25 (78)	14 (78)		
Neck	3 (9)	0		
Body/tail	4 (13)	4 (22)		
Macroscopic pattern			0.757	0.70
Round/Oval	20 (66)	13 (72)		
Irregular	12 (34)	5 (28)		
Margin			0.168	0.80
Well-defined	5 (16)	1 (6)		
Moderately-defined	16 (50)	6 (33)		
Ill-defined	11 (34)	11 (61)		
Distal atrophy ^a^			0.239	0.79
Present	13 (41)	11 (61)		
Absent	19 (59)	7 (39)		
T1WI signal intensity			0.055	0.81
isointense	3 (9)	6 (33)		
Hypointense	29 (91)	12 (67)		
T2WI signal intensity			0.730	0.79
Hyperintense	27 (78)	15 (83)		
isointense	5 (22)	3 (17)		
DWI signal intensity			0.019	0.66
Hyperintense	22 (69)	5 (28)		
isointense	8 (25)	11 (61)		
Hypointense	2 (6)	2 (11)		
Enhancement of mass				
The pancreatic phase			0.382	0.76
Homogeneous	13 (41)	10 (56)		
Heterogeneous	19 (59)	8 (44)		
The portal phase			0.036	0.64
Homogeneous	14 (44)	14 (78)		
Heterogeneous	18 (56)	4 (22)		
The delayed phase			0.016	0.68
Homogeneous	15 (47)	11 (83)		
Heterogeneous	17 (53)	7 (17)		
Elevated CA 19-9			<0.01	-
Present	28(88)	5(28)		
Absent	4(12)	13(72)		
Elevated IgG4			0.047	-
Present	0	^b^3(17)		
Absent	32(100)	15(83)		

a Lesions located in the edge of the pancreatic tail were excluded.

b The underlying causes of the 3 FPs with elevated IgG4 were autoimmune pancreatitis.

**Table 2:** Frequencies of the tumor clinical stage and pathological grade of pancreatic cancer

	PC (n, %)
T stage	
1	1 (3)
2	8 (25)
3	23 (72)
4	0
N stage	
0	20 (63)
1	12 (37)
M stage	
0	32 (100)
1	0
TNM staging	
I A	2 (6)
IB	5 (16)
IIA	13 (41)
IIB	12 (37)
III	0
IV	0
Histology grade	
Well-differentiated	2 (6)
Moderately differentiated	18 (56)
Poorly differentiated	12 (38)

### Qualitative analysis of MRI

With regard to the MRI features of PC and FP, there were no significant differences in location, macroscopic pattern, margin, distal atrophy, T1WI signal intensity, T2WI signal intensity, and homogeneity of enhancement in pancreatic phase. However, PC was more likely to show a heterogeneous enhancement, while FP was more common to display a homogeneous enhancement during the portal (*P* < 0.036) and delayed (*P* < 0.016) phases. Also, a statistically significant difference was observed in DWI signal intensity between PC and FP. Lesion signal intensity compared with the adjacent pancreatic parenchyma on DWI(*P* = 0.01): as for PC cases, 22/32 (69%) appeared hyperintense, 8/32 (25%) isointense, and 2/32 (6%) hypointense, while 11/18 (61%) isointense, 5/18 (28%) hyperintense, and 2/18 (11%) hypointense for FP cases. Elevated CA19-9 presented more frequently in PC than in FP (*P* < 0.01). Elevated IgG4 only presented in 3 FP (*P* = 0.047) in which the underlying causes were all autoimmune pancreatitis.

**Figure 1:**
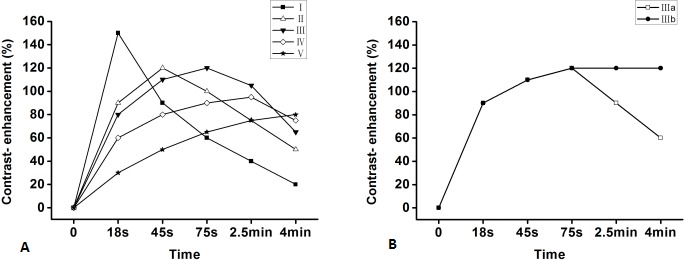
Patterns of the TIC from DCE MRI of the pancreas According to the time of a peak (18s, 45s, 75s, 2.5min, 4min after bolus injection of contrast material), namely, type-I, II, III, IV, V, respectively **A.** Then, according to the part of the TIC profile after the peak time, the type of the masses were classified into two subtypes, subtype-a (washout, the contrast enhancement decrease more than 10% of the peak time) and subtype-b (plateau, the contrast enhancement does not decrease more than 10% after the peak time) **B.**

### Semi-quantitative analysis of DCE-MRI

**Figure 2:**
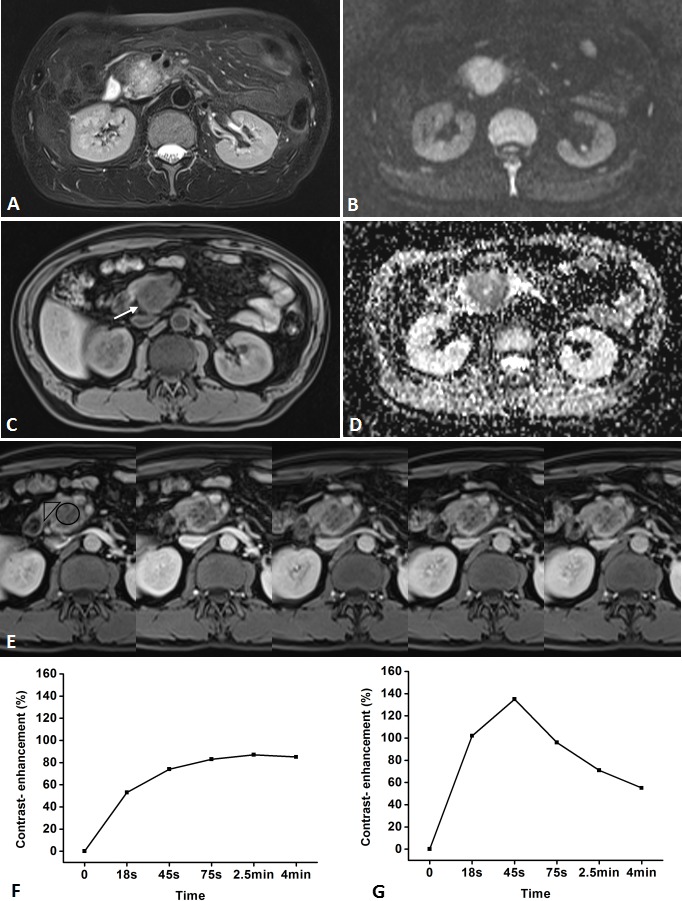
Representative pancreatic T2-weighted image (A), DWI with a b value of 600 s/mm^**2**^ (B), T1-weighted image (C), ADC map (D), DCE-MR images (E), and TIC profiles (F, G) in a 49-year-old man with pancreatic carcinoma in the head of pancreas (white arrow) DCE-MR images: 18s, 45s, 75s, 2.5 and 4 min aftercontrast injection with constant gray scale. The ROIs of mass and non-mass adjacent parenchyma (NAP) indicated with black circle and black triangle. Pancreatic mass demonstrates type-IV b TIC which shows a slow, gradually increasing enhancement pattern followed by a plateau, while NAP demonstrates type-II aTIC which shows a rapidly increasing then gradually decreasing enhancement pattern. DWI shows pancreatic mass is clearly seen as hyperintense with a well-defined margin.

**Figure 3:**
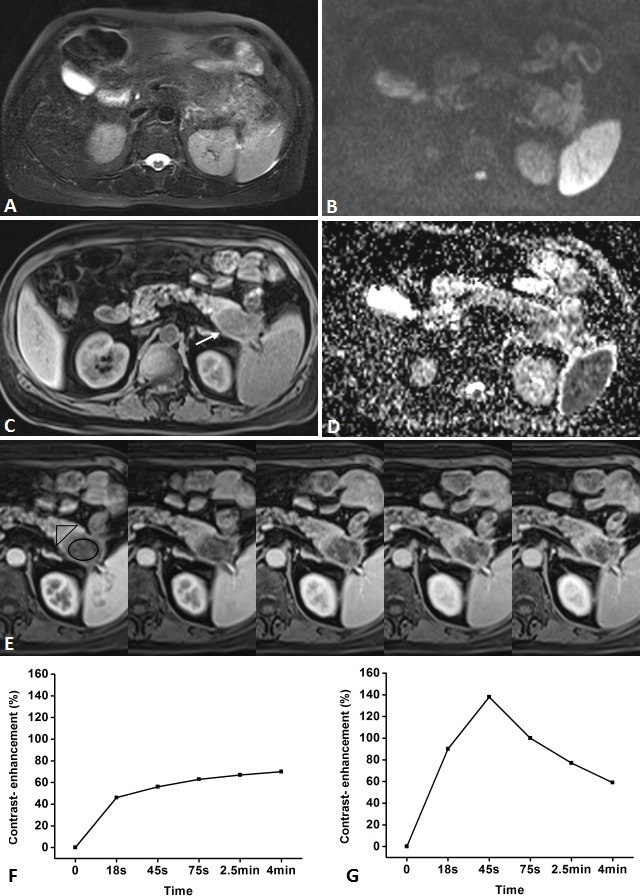
Representative pancreatic T2-weighted image (A), DWI with a b value of 600 s/mm^**2**^ (B), T1-weighted image (C), ADC map (D), DCE-MR images (E), and TIC profiles (F, G) in a 62-year-old man with pancreatic carcinoma in the tail of pancreas (white arrow) DCE-MR images: 18s, 45s, 75s, 2.5 and 4min aftercontrast injection with constant gray scale. The ROIs of mass and non-mass adjacent parenchyma (NAP) indicated with black circle and black triangle. Pancreatic mass demonstrates type-VTIC which shows a slow, gradually increasing enhancement pattern, while NAP demonstrates type-II aTIC which shows a rapidly increasing then gradually decreasing enhancement pattern. DWI shows pancreatic mass is clearly seen as isointense withmoderately-defined margin.

As summarized in Table 3, PC demonstrated type-V (*n* = 10), type-IV b (*n* = 8), type-III b(*n* = 7), type-IV a(*n* = 5) or type-III a (*n* = 2) TIC, which reveal most TIC trend of a slow, gradually increasing enhancement pattern (Figures 2, 3). In contrast, the NAP of PC showed type-II a(*n* = 19), type-II a (*n* = 6), type-III a (*n* = 4) or type-IV a (*n* = 3)TIC. There is significant statistical difference between mass and NAP of PC (*P* < 0.01).

**Table 3:** The comparison of the TIC of the mass and NAP between PC and FP

	PC		FP		*P* Value		^b^ICC	
	Mass	^a^NAP	Mass	NAP	Mass	NAP	Mass	NAP
TIC pattern							0.897	0.916
I a	0	19	3	4	0.037	0.018		
I b	0	0	0	0	-	-		
II a	0	6	6	8	0.001	0.099		
II b	0	0	0	0	-	-		
III a	2	4	3	6	0.324	0.183		
III b	7	0	3	0	0.391	-		
IV a	5	3	2	0	1.000	0.544		
IV b	8	0	1	0	0.036	-		
V	10	0	0	0	0.008	-		

a NAP, non-tumor adjacent pancreatic parenchyma

b ICC, intra-class correlation coefficient

**Figure 4:**
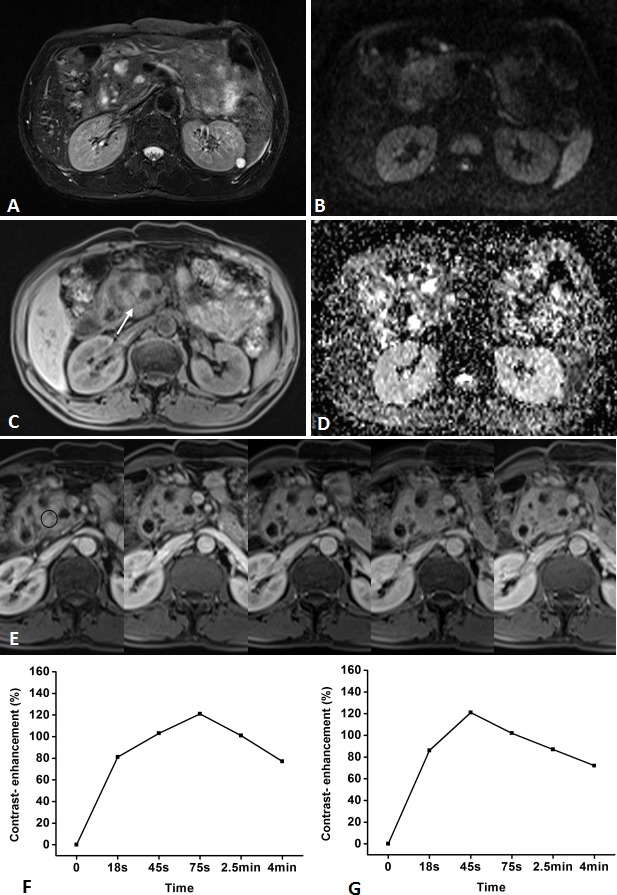
Representative pancreatic T2-weighted image (A), DWI with a b value of 600 s/mm^**2**^ (B), T1-weighted image (C), ADC map (D), DCE-MR images (E), and TIC profiles (F, G) in a 55-year-old man with mass-forming chronic focal pancreatitis in the head of pancreas (white arrow) DCE-MR images: 18s, 45s, 75s, 2.5 and 4min after contrast injection with constant gray scale. The ROIs of mass indicated with black circle and non-mass adjacent parenchyma (NAP) was located in pancreatic body. Pancreatic mass demonstrates type-III a TIC which shows a gradual increase followed by a more slowly decreasing enhancement pattern, while NAP demonstrates type-II a TIC which shows a relatively rapid increasing then gradually decreasing enhancement pattern. DWI shows pancreatic mass is clearly seen as isointense with ill-defined margin.

**Figure 5:**
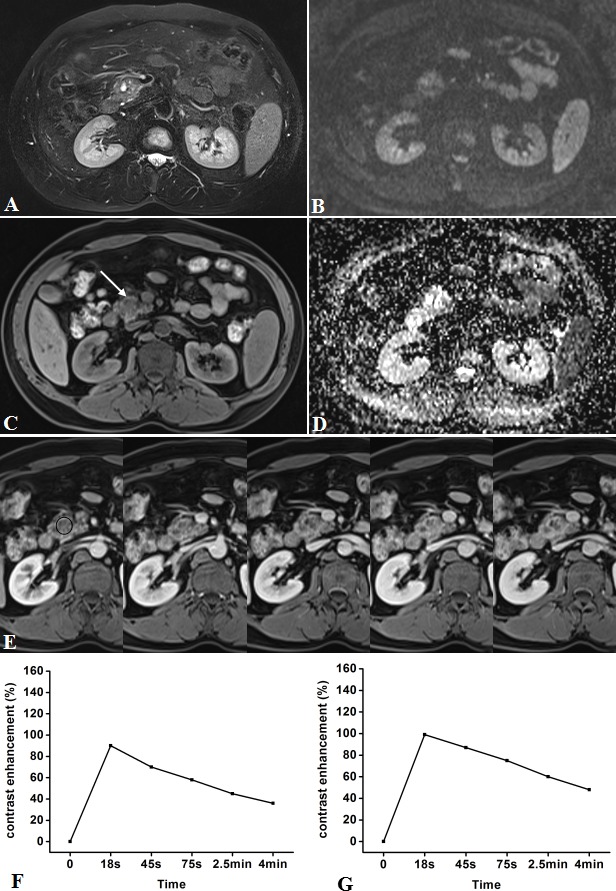
Representative pancreatic T2-weighted image (A), DWI with a b value of 600 s/mm^**2**^ (B), T1-weighted image (C), ADC map (D), DCE-MR images (E), and TIC profiles (F, G) in a 43-year-old man with mass-forming chronic focal pancreatitis in the head of pancreas (white arrow) DCE-MR images: 18s, 45s, 75s, 2.5 and 4min after contrast injection with constant gray scale. The ROIs of mass indicated with black circle and non-mass adjacent parenchyma (NAP) was located in pancreatic body. Pancreatic mass and NAP all demonstrates type-I a TIC which shows a rapidly increasing then gradually decreasing enhancement pattern. DWI shows pancreatic mass is clearly seen as isointense/mild-hyperintense with ill-defined margin.

FP demonstrated type-II a (*n* = 6), type-I a (*n* = 3), type-III a(*n* = 3), type-III b(*n* = 3), type-IV a(*n* = 2) or type-IV b (*n* = 1) TIC, which reveal most TIC trend of a gradual increase followed by a more slowly decreasing enhancement pattern (Figures 4, 5). In contrast, the NAP of FP showed type-II a(n = 8), type-III a (*n* = 6) or type-I a(*n* = 4) TIC. There is no statistical difference between massand NAP of FP (*P* = 0.081).

The prevalent TIC profiles differed in the PC and FP groups in that PC showed the type-I or subtype-b profile (25 of 32, 80%) and FP showed the type-I, type-II or subtype-a profile (14 of 18, 78%). The type-V TIC was only recognized in PC group (*P* = 0.008), while type-I and type-II only in FP group (*P* = 0.037, 0.001). Furthermore, the TIC of PC frequently depicted as slower increase to the peak than FP.

### Quantitative analysis of DWI

**Figure 6:**
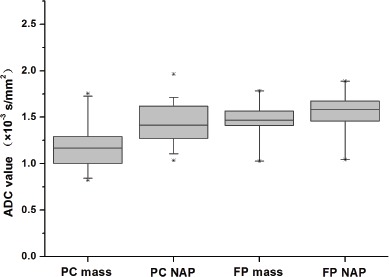
Boxplots of the ADC value of the mass and non-mass adjacent parenchyma (NAP) of pancreatic carcinoma (PC) and mass-forming focal pancreatitis (FP)

The results of quantitative analysis in DWI are summarized in Table 4 and Figure 6. The mean diameter for all masses of PC was 3.3 ± 1.4cm (mean ± standard deviation), with a range in diameter of 0.9-5.9 cm, and without any statistical difference between PC and FP (4.1 ± 1.5cm, 2.0-6.3cm) (*P* = 0.052). The mean ADC value ± standard deviations (×10^−3^mm^2^/s) of masses are lower in PC than FP (1.17 ± 0.23, 1.47 ± 0.18, respectively, and *P* < 0.01), while no statistical difference of NAP was revealed between PC and FP at DWI (1.43 ± 0.22, 1.55 ± 0.22, respectively, and *P* = 0.098). A significant difference was also found in the mass to NAP contrast ratio of ADC between PC and FP (0.37 ± 0.19, 0.16 ±0.09, respectively, and *P* < 0.01).

**Table 4:** The comparison of ADC value and ADC contrast ratio between PC and FP

	PC	FP	*P* Value	^b^ICC
**ADC Value**				
Mass	1.17 ± 0.23	1.47 ± 0.18	< 0.01	0.825
^a^NAP	1.43 ± 0.22	1.55 ± 0.22	0.098	0.837
ADC contrast ratio	0.37 ± 0.19	0.16 ±0.09	< 0.01	0.751

a NAP, non-mass adjacent pancreatic parenchyma

b ICC, intraclass correlation coefficient

### ROC analysis of DCM-MRI and DWI

**Figure 7:**
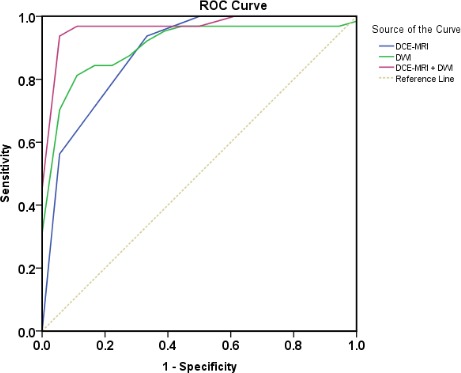
Receiver operating characteristic curves used to evaluate diagnostic performance of the DCE-MRI, DWI, and combined imaging sets, respectively (area under ROC: 0.885 ± 0.052, 0.913 ± 0.041, and 0.979 ± 0.018) for differentiation between pancreatic carcinoma and mass-forming focal pancreatitis

As summarized in Figure 7, in the differentiation between PC and FP, ROC showed the AUC was significantly improved in the combined image sets (0.979 ± 0.018; 95% CI: 0.943, 1.000)of DCE-MRI set and DWI set compared with DCE-MRI set (0.885 ± 0.052; 95% CI: 0.784, 0.986) and DWI set (0.913 ± 0.041; 95% CI: 0.832, 0.994) alone. In addition, setting the cutoff value of ADC 1.3036, ADC contrast ratio 0.2625, and TIC 3.5, the combined image set of DCE-MRI and DWI (96.9%, 94.4%, 96.0%) yielded a better sensitivity, specificity, and diagnostic accuracy than the DCE-MRI (93.8%, 66.7%, 84.0%, *P* < 0.01) or DWI alone (84.4%, 88.9%, 86.0%, *P* < 0.01).

**Figure 8:**
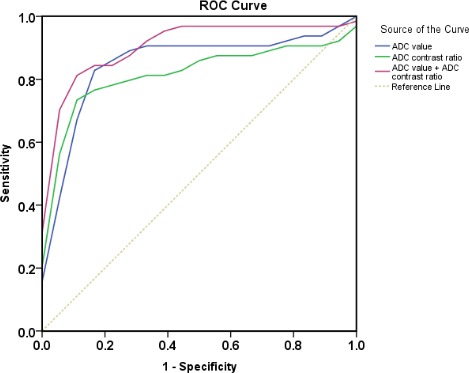
Receiver operating characteristic curves used to evaluate diagnostic performance of ADC value, ADC contrast ratio and combined of them, respectively (area under ROC: 0.852 ± 0.059, 0.821 ± 0.061, and 0.913 ± 0.041) for differentiation between pancreatic carcinoma and mass-forming focal pancreatitis

As summarized in Figure 8, in the differentiation between PC and FP, ROC showed the AUC was significantly improved in combined of ADC value and ADC contrast ratio (0.913 ± 0.041; 95% CI: 0.832, 0.994) compared with ADC value (0.852 ± 0.059; 95% CI: 0.701, 0.941) and ADC contrast ratio (0.821 ± 0.061;95% CI: 0.738, 0.967) alone. In addition, the sensitivity of ADC value combined with ADC of mass to NAP contrast ratio was higher than that of ADC value and ADC of mass to NAP contrast ratio alone (84.4%, 81.3%, 71.9%; *P* < 0.01), despite there is no significant difference on statistics between the specificities of them (88.9%, 88.9%, 94.4%; *P*>0.05).

### Interobserver agreement

Good or excellent interobserver agreement was established during the qualitative evaluation of conventional MRI fingdings, whereas excellent intraobserver agreement was reached based on the measurement of ADC and DCE-MRI quantitative and semi-quantitative parameters. Kappa values for qualitative assessment of the conventional MRI features were listed in Table 1, and the ICCs for the measurement of ADC and DCE MRI parameters were listed in Tables 3, 4.

## DISCUSSION

In differentiating the focal pancreatic masses with DCE-MRI between PC and FP, results from the present study demonstrated that PC exhibits a specific TIC in comparison with FP. The type-V TIC was a peculiar profile to PC since no FP demonstrated this TIC type. A representative TIC profile pattern of the PC was either type-V or subtype-b (25/32, 80%) in the mass. FP showed the type-I, type-II or subtype-a profile (14/18, 78%), and the type-I and type-II TIC was only recognized in FP group. In this study, since the TIC typing was based on the time of reaching the peak, the TIC of PC revealed a slower rise to a peak than FP. These were consistent with the previous studies that pancreatic ductal carcinoma are characterized by a very tiny microvascular structure with a high permeability resulting in hypovascular tumoral phenotype [17, 18]. Therefore, PC showed a slow gradual enhancement pattern, whereas FP showed an earlier and more obvious enhancement followed by a slow decreasing pattern compared with PC. Moreover, PC was more likely to show a heterogeneous enhancement; in contrast, FP demonstrated a homogeneous enhancement during the portal and delayed phases.

The differential diagnosis of PC and FP is a common challenge in clinical context [19–21]. Because this confusion may lead to surgical treatment for benign diseases or ignorance of a curable PC, the differential diagnosis of these two diseases is crucial. In the present study, the TIC profile of PC overlapped with that of FP, i.e., type-III and type-IV TIC. The type-III and type-IV TIC accounted for 28% (9/32) and 40% (13/32) of PC, while 33% (6/18) and 17% (3/18) of FP. However, in the overlap of TIC types, subtype-b (plateau) profile accounted for 68% (15/22) of PC, while subtype-a (washout) profile 56% (5/9) of FP. This is probably because PC with a relatively large extravascular extracellular space and abundant fibrous stroma can retain contrast materials for a longer time, while FP with relatively small extravascular extracellular space, will retain contrast materials more transiently [22, 23]. Therefore, different TIC type and subtype of PC and FP may provide useful information in reaching the correct diagnosis.

The prior studies [23–25] have unveiled that the pathological hypovascularity of chronic pancreatitis and PC were both associated with parenchymal fibrosis, a decreased blood vessel density and blood flow of pancreas. The micro vessel density, the quantity of aqueous protein, and the extent of fibrosis in the pancreas as well as the difference in the mass-to-pancreatic parenchyma contrast, may contribute to the enhancement degree of pancreatic masses on MRI. To precisely clarify the pancreatic masses based on the DCE-MRI, a qualitative and quantitative evaluation were required for the changes in microcirculation of relevant pancreas in the process of intratumoral angiogenesis and the occlusion of the small vessels by fibrosis or cancer cells.

As for optimization of b value, the use of 600 s/mm^2^ in DWI was suggested for differentiating the benign from the malignant lesions for the abdomen[26]. In this study, the mean ADC value of masses is lower in PC than FP, while no statistical difference of NAP was revealed between PC and FP at DWI. However, relative ADC values may be more capable of assessing the tissue vasculature [14, 27]. Koc, Z. and G. Erbay found that the lesion ADC to normal parenchyma ADC ratio is more accurate than utilizing lesion ADC only for the differentiation [26]. In the present study, the unaffected normal spleen parenchyma were chosen as a more reliable reference in contrast to NAP which may present inflammation, fibrosis as well as acinar cell loss [28],. We found that the ADC value combined with mass-to-NAP ratio of ADC value has a better sensitivity than the mass ADC value alone, although there is no significant difference in the specificity between them. The results of this research and some previous reports [29–31] demonstrate that DWI can be valuable for detection of pancreatic lesions as well as differentiation of PC from FP.

Sugiyama et al. [32] mainly focused on morphological differences of MRI features between focal AIP and PC. Our study is a comprehensive application of multi-parametric MRI, which includes not only morphological and signal features of conventional MRI but also DCE-MRI and DWI. Vijayakumar et al. [33] and Kamisawa et al. [34] reported that ADC values can improve the ability to distinguish autoimmune pancreatitis (AIP) from PC, and the ADC values were significantly higher in PC patients or in individuals with a normal pancreas than in AIP. Although cancer cell infiltration with desmoplastic stroma is the typical histopathological feature of pancreatic cancer, the cellularity of massive lymphoplasmacytic infiltration in AIP is more abundant than that of PC [34]. AIP with increased cellularity and edematous changes may be vulnerable to produce lower ADC values than PC [34]. In our study, the underlying causes of 18 FP included alcoholic (*n* = 10), idiopathic (*n* = 3), autoimmune (*n* = 3), gallstone (*n* = 1), and pancreatic divisum (*n* = 1). Therefore, there are various underlying diseases of mass-forming focal pancreatitis, and AIP is just one special type of chronic pancreatitis, which may explain the differences existing between the present study and the above-mentioned ones.

In general, the limited diffusion in tumor tissue has been ascribed to hypercellularity. However, diffusivity is intrinsically affected by the extracellular fibrosis, intracellular spaces, and glandular structure [35]. The DWI signal intensity and the ADC values of PC on DWI are dependent on the cellularity and amount of tissue fibrosis [36]. FP is featured by moderate to severe inflammatory process as well as the progressive destruction and fibrosis of pancreatic parenchyma on histopathology [37]. FP was of similar homegeneous signal intensity or hyperintense to remaining pancreas while PC could be recognized from remaining pancreas because of hyperintense signal comparing to the remaining pancreas on DWI with b = 600s/mm^2^.

Our ROC analysis showed that qualification analysis of TIC in DCE-MRI acquired a sensitivity of 93.8% and a specificity of 66.7% for differentiating PC from FP, the quantification analysis of ADC value and ADC contrast ratio in DWI obtained 84.4%, 88.9%, and the combined DCE-MRI with DWI obtained 96.9%, 94.4%, indicating a benefit of combined image. However, there's an overlap in ADC values between PC and FP in some cases. This might result from the variable tumoral components in PC and the histopathological features of the FP. Consequently, the relative lower degree of fibrosis might result in increased water diffusion to some extents in PC [36]. FP may contain variable proportions of fibrosis and inflammation, which may explain variations among studies and overlap of ADC values for PC and FP [38]. The intravoxel incoherent motion (IVIM) technique with multi-b acquisition was not performed in the present study, thus, an estimate of the perfusion fraction (f) and the perfusion free diffusion parameter (D) on DWI were not acquired [39, 40]. It is worth noting, however, that TIC of DCE-MRI was used in our study, which assumed a qualitative assessment of the changes in pancreatic hemodynamics. Certainly, we believe that the IVIM model could be applied to differentiate PC and FP.

Several limitations of this study should be documented. First, the retrospective design might have caused some selection and verification bias, because, in the study, MRI of the pancreas is usually performed for patients who are suspected of having PC or FP and when they are considered possible surgical candidates. Second, our results were obtained from a limited cohort study, especially for the FP group, just reflecting our preliminary experience. It would be necessary to perform larger studies to validate our findings. Third, because of a potential sampling error, a wrong classification of pancreatic mass lesions may occur from an FNA biopsy. When early PC is buried by an inflammatory mass, a missed diagnosis may be diminished by extending the follow-up period for FP. More prospective studies are warranted to testify the results of this study.

In conclusion, the TIC from DCE-MRI and ADC value mass-to-NAP contrast ratio of ADC value from DWI have been revealed to offer valuable data for distinguishing PC from FP, and the combined analysis of DCE-MRI and DWI can achieve a higher sensitivity, specificity, and diagnostic accuracy. Therefore, these imaging techniques may enable elimination of a dispensable pancreatic surgery for FP and a correct diagnosis of PC preoperatively.

## MATERIALS AND METHODS

### Patients

This retrospective study was approved by the Institutional Review Board of our hospital, and the requirement for informed consent for the patient data review was waived. Cases for the PC and FP groups were recruited from by reviewing the medical records from January 2011 to February 2014 of patients who had undergone clinical DCE-MRI and DWI and in whom a pancreatic mass was suspected. The PC group was composed of 32 consecutive patients (18 males, 14 females, age range 44–77 years, mean 59.5 ± 9.3 years) with surgical and histological proof of PC through Whipple procedure (*n* = 29) and endoscopic retrograde cholangiopancreatography (ERCP)guided fine needle aspiration(FNA) (*n* = 3). The FP subject group consisted of 18 consecutive patients (12 males, 6 females, age range 14–72 years, mean 47.6 ± 12.4 years) in whom the diagnosis of FP was determined by surgical resection (*n* = 8), or ERCP-guided FNA (*n* = 6) or endoscopic ultrasound-guided FNA (*n* = 4).

### Imaging protocol

MRI was performed with a 3-T whole-body clinical MRI scanner (Signa HDxt; GE Healthcare, Milwaukee, WI, USA) equipped with 8-channel TORSOPA coils. We used a fat-suppressed liver acquisition with volume acceleration (LAVA) sequence included unenhanced, enhanced T1WI (TR/TE, 5.8/2.6msec; flip angle, 10°; section thickness, 3mm; no intersection gap; matrix, 142×256; number of excitation, 1; field of view, 300×400mm), and unenhanced T2WI (TR/TE, 7000/104msec; flip angle, 125°; section thickness, 5mm; intersection gap, 10%; matrix, 173×384; number of excitation, 2; field of view, 300×400mm). The dynamic series included 6 individual dynamic phases of images, acquired before and 18s (early arterial phase), 45s (late arterial/pancreatic phase), 75s (portal phase), 2.5min (delayed phase), and 4min (late delayed phase) after the rapid bolus injection of 0.2 mmol of Gd-DTPA (Magnevist; Beilu, Beijing, China)/kg of body weight. The contrast agents were administered intravenously at 3 ml/s before flushing with 20 ml saline solution was conducted.

DWI was implemented by using a single-shot, spin-echo, echo-planar imaging (EPI) sequence with DWI gradients (b value, 0 and 600s/mm^2^) utilized in three orthogonal directions. The related parameters of DWI were as follows: axial imaging; fat saturation, chemical shift imaging; acquisition model, breathe triggering; parallel imaging technique, array spatial sensitivity encoding technique (ASSET; GE Healthcare, Milwaukee, WI, USA); TR, 5000 ms; TE 78ms; slice thickness/space, 5/1 mm; matrix, 293×360; field of view, 42 cm; number of excitation, 3.

### Image analysis

Two radiologists with 9- or 15-year experience in abdominal imaging who were not informed of histopathological results performed the MRI features of PC and FP in consensus. The disputes between the radiologists were resolved by consultation with a third experienced radiologist specializing abdominal imaging for 18 years. The original MRI data were loaded onto a dedicated workstation (GE Medical Systems), and the regions of interest (ROIs) such as pancreatic masses and NAP were determined based on the signal-intensity difference between the ROIs and background in diffusion-weighted and contrast-enhanced MR images to retrieve diffusion and perfusion parameters, respectively, by the two radiologists. The ROI was lesion-size-dependent and no less than 100 mm^2^. The signal intensity and ADC value were recorded as the average of three separately measured ROI on each image. The following items were analyzed for each mass: lesion size (longest diameter in centimeters), location of lesion (pancreatic head, neck, body, or tail), shape (round, oval, or irregular), margin (well-defined, moderately-defined, or ill-defined), presence of parenchymal atrophy, T_1_WI signal intensity (hypointense, isointense, and hyperintense), T_2_WI signal intensity, DWI signal intensity, and homogeneity of enhancement in pancreatic, portal, and delayed phase (homogeneous, heterogeneous).

The pancreatic TIC was then generated as a percentage increase in the signal intensity (SI), according to the following enhancement formula: (SI_post_-SI_pre_)/SI_pre_ × 100%, where SI_pre_ and SI_post_ represent the pre- and post-contrast SIs, respectively. The patterns of pancreatic TIC were classified into 5 types (Figure 1A): type-I, characterized by a rapid rise to a peak (18 s after administration of contrast material) followed by a rapid decline; type-II, with a relatively rapid rise to a peak (45 s after injection of contrast material) followed by a slow decline; and type-III, IV or V, with an even slower rise to a peak (75s, 2.5 or 4 min after the injection of contrast material). Then, according to the profile of TIC after the peak time, the type of the masses were classified into two subtypes (Figure 1B), subtype-a (washout, the contrast enhancement decrease more than 10% after reaching the peak time) and subtype-b (plateau, the contrast enhancement decrease less than 10% after reaching the peak time). A retrospective review of the preoperative pancreatic MRI study and pancreatic histology was performed, and the patterns of TIC from DCE-MRI depicted at the 3 parts of the pancreas were then compared with the pertinent pathological consequences in each case.

In DWI analysis, ADC maps were created by using the signals acquired at different b values (0, 600 s/mm^2^) on the interactive workstation with dedicated software. For all the patients, the ADC values of pancreatic focal mass lesions and NAP were measured by one investigator with an ROI, which were dependent on lesion size and no less than 100 mm^2^. The mass-to-NAP contrast ratio of ADC values was calculated as follows: | (SI_T_ – SI_P_) | / SI_S_, where SI_T_ is signal intensity of pancreatic mass, SI_P_ is signal intensity of NAP, and SI_S_ is signal intensity of spleen. The ADC of the spleen was used for the normalization of ADC values.

DCE-MRI and ADC are combined for analysis as follows. First, the 5 types of TIC (I, II, III, IV, and V) are scored 1, 2, 3, 4, and 5 respectively, and additionally the subtype b will get another 1 score and subtype a will not, for example, type IIb is scored 3 (2+1). Second, the mean ADC value and mass-to-pancreas contrast ratio of ADC value are combined. Finally, we combined TIC of DCE-MRI after scoring with integrated ADC which was obtained with the combination of the absolute and relative ADC.

### Statistical analysis

Intergroup comparisons between PC and FP were conducted by using Fisher's exact test or Pearson Chi-Square for categorical variables and the Student's t test for numeric variables. The TIC types were compared by using Fisher's exact test and the ADC value and the mass-to-NAP contrast ratio of ADC value were compared by using the Student's t test. The diagnostic performance of TIC and ADC quantification in differentiating PC from FP was assessed with receiver operating characteristic (ROC) analysis, which provided the sensitivity and specificity of DCE-MRI or DWI alone and the combined ones. The McNemar test was used for comparison of sensitivity and specificity of DCE-MRI or DWI alone and the combined ones. The interobserver agreement for qualitative MRI features was evaluated by using Kappa analysis. The interobserver agreement for quantitative DWI and DCE-MRI parameters was evaluated by using the intra-class correlation coefficient (ICC) and implementing a two-way ICC with a random rater assumption. The ICC and kappa value ranges 0–1.00, with values closer to 1.00 representing better reproducibility. They were stratified as follows: (< 0.40, poor; 0.41–0.60, moderate; 0.61–0.80, good; and >0.81, excellent). The combined data of DCE-MRI and ADC were analyzed by using receiver operating characteristic curve (ROC). P values less than 0.05 were considered significant, and data were presented as mean ± standard deviation with their range in brackets. All analyses were performed by using SPSS, version 13.0.1 (SPSS Inc., Chicago, IL, USA).
